# Laccases as Effective Tools in the Removal of Pharmaceutical Products from Aquatic Systems

**DOI:** 10.3390/life14020230

**Published:** 2024-02-06

**Authors:** Daniela Chmelová, Miroslav Ondrejovič, Stanislav Miertuš

**Affiliations:** 1 Department of Biotechnology, Faculty of Natural Sciences, University of Ss. Cyril and Methodius, J. Herdu 2, SK-91701 Trnava, Slovakia; daniela.chmelova@ucm.sk (D.C.); miroslav.ondrejovic@ucm.sk (M.O.); 2 ICARST n.o., Jamnického 19, SK-84101 Bratislava, Slovakia

**Keywords:** enzyme, remediation, wastewater, laccases, pharmaceuticals, degradation, biotransformation

## Abstract

This review aims to provide a comprehensive overview of the application of bacterial and fungal laccases for the removal of pharmaceuticals from the environment. Laccases were evaluated for their efficacy in degrading pharmaceutical substances across various categories, including analgesics, antibiotics, antiepileptics, antirheumatic drugs, cytostatics, hormones, anxiolytics, and sympatholytics. The capability of laccases to degrade or biotransform these drugs was found to be dependent on their structural characteristics. The formation of di-, oligo- and polymers of the parent compound has been observed using the laccase mediator system (LMS), which is advantageous in terms of their removal via commonly used processes in wastewater treatment plants (WWTPs). Notably, certain pharmaceuticals such as tetracycline antibiotics or estrogen hormones exhibited degradation or even mineralization when subjected to laccase treatment. Employing enzyme pretreatment mitigated the toxic effects of degradation products compared to the parent drug. However, when utilizing the LMS, careful mediator selection is essential to prevent potential increases in environment toxicity. Laccases demonstrate efficiency in pharmaceutical removal within WWTPs, operating efficiently under WWTP conditions without necessitating isolation.

## 1. Introduction

The global pharmaceutical market has witnessed substantial growth, with a projected value of USD 1.48 trillion by 2022 [1]. Approximately one-third of pharmaceuticals in America end up as waste [2], leading to their detection in wastewater, groundwater, surface water, and even drinking water. Their presence raises concerns due to the documented negative environmental impact [3] and the potential emergence of new diseases through their untargeted administration [4,5,6,7,8].

Various methods, including chemical, physicochemical, or physical methods like membrane processes, advanced oxidation processes (e.g., ozonation, UV photolysis or UV/H_2_O_2_) adsorption, are employed for pharmaceutical removal. However, these methods present drawbacks such as the generation of toxic by-products in advanced oxidation processes [9,10,11,12,13], the disposal challenges associated with concentrated waste in membrane processes [14] or the difficulty of regenerating sorbents [15,16]. Biological processes, especially enzymatic processes, offer numerous advantages over physicochemical or alternative biological processes. They are inherently safer, generate less sludge, demand lower energy input, and operate without the need for additional nutrients. The resulting by-products are generally less or non-toxic, and these processes prove effective even at very low pollutant concentrations in wastewater [17,18]. Oxidoreductases, such as laccases, stand out as predominant enzymes in these processes [19,20,21,22], owing to their versatility and an active center accommodating a broad range of substrates [23]. Laccases offer the added advantage of requiring only molecular oxygen for catalyzing reactions. Moreover, their production is widespread across organisms like bacteria, fungi, and plants, with white-rot fungi, in particular, being highly efficient producers [24,25,26,27]. Fungal laccases exhibit high activity and interesting properties that are useful in bioremediation processes, such as substrate specificity, pH, and the optimum temperature of the enzyme, which are also useful in wastewater treatment plants (WWTPs) for their potential use in pollutant removal.

This review aims to provide an up-to-date overview of the potential use of laccase in removing pharmaceutical products encompassing analgesics, antibiotics, antiepileptics, antirheumatic drugs, cytostatics, hormones, anxiolytics, and sympatholytics. The focus lies on studies utilizing free purified laccase, where the impact on pharmaceutical substances can be directly attributed to laccase. This involves detailing pharmaceutical product concentrations in wastewater, characterizing degradation or biotransformation products and assessing their toxic effects before and after enzyme-catalyzed reactions. 

## 2. Laccase

**Figure 1:**
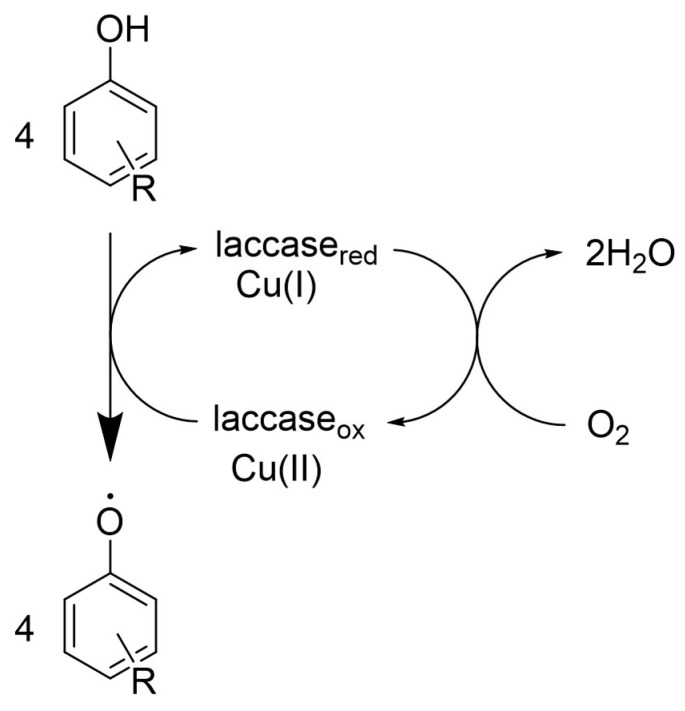
General laccase reaction mechanism.

Laccase (EC 1.3.1.10) is an enzyme belonging to the class of oxidoreductases. It catalyzes the four-electron oxidation of a substrate in the presence of molecular oxygen as a co-substrate to form water (Figure 1).

Laccases exhibit variability in redox potential depending on their source, with the lowest redox potential observed in laccases isolated from plants, followed by bacteria and fungi [28]. Notably, laccases derived from white-rot fungi have a high redox potential, enabling them to effectively degrade challenging substances, including pharmaceuticals [29,30]. Laccases catalyze the direct oxidation of both phenolic and nonphenolic substrates [31,32,33]. For substances with a high redox potential that are challenging to degrade, enhancing degradation efficiency is achievable by employing low-molecular-weight substances known as redox mediators within the laccase mediator system (LMS). Redox mediators facilitate electron transfer between the substrate and the enzyme between the substrate and the enzyme [34,35]. In the context of degrading or biotransforming pharmaceutical products using laccase, the literature commonly features the use of the LMS, particularly with bacterial and fungal laccases. A summarized overview of these laccases and their properties is provided in Table 1.

The laccase-catalyzed degradation of a compound follows a process wherein an electron is transferred from the compound to the laccase. This electron is stabilized in the enzyme’s active center to copper atom. Subsequently, the copper center undergoes regeneration through a coupled reaction with molecular oxygen, resulting in the formation of water. The degree of degradation is significantly and positively influenced by the structural properties of the oxidized compound. Factors such as the presence of an aromatic or heterocyclic ring, the presence of electron-donor groups, coupled bonds, and easily oxidizable substituents play crucial roles in determining the extent of degradation [51].

## 3. Pharmaceutical Products

**Figure 2:**
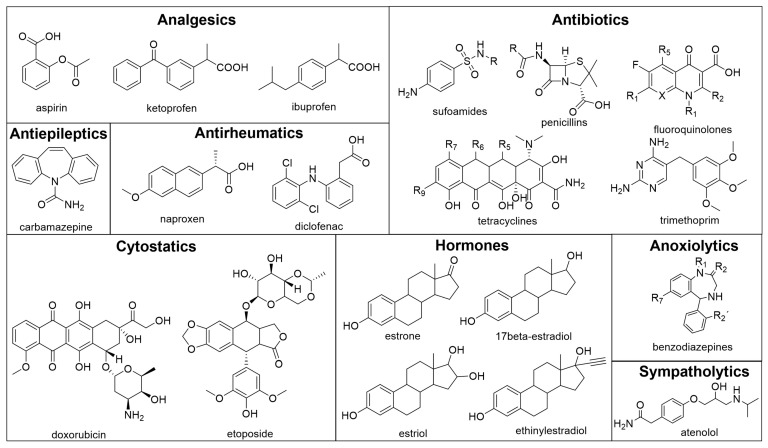
Pharmaceuticals tested as potential substrates for laccase.

The removal of pharmaceutically active substances by enzymes such as laccases has received considerable attention, especially in recent years. Purified laccases were first tested on organic dyes and structurally simple substrates [19,20,21,22]. The current research deals with the use of laccase for the removal of pharmaceutically important compounds belonging to drug groups, such as analgesics, antibiotics, antiepileptics, antirheumatic drugs, cytostatics, hormones, anxiolytics and sympatholytics. The structures of the drugs tested are shown in Figure 2.

### 3.1. Analgesics

Analgesics, encompassing both non-opioid and opioid varieties, serve as pain-relieving medications. In this category, laccase has been tested against nonsteroidal anti-inflammatory drugs, specifically aspirin and ketoprofen. 

**Figure 3:**
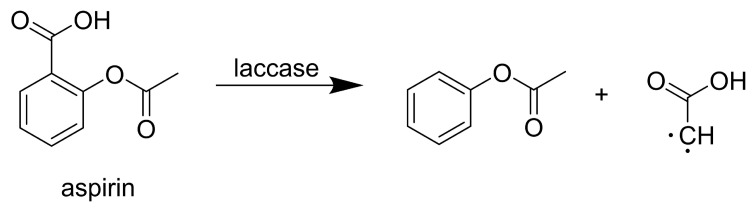
Biotransformation of aspirin by laccase from *Y. enterocolitica* expressed in *E. coli* [40]. Copyright (2024) with permission from Elsevier.

Aspirin (acetylsalicylic acid), a non-steroidal anti-inflammatory drug used to manage pain and fever, poses a threat to the aquatic ecosystem due to its excessive use, serving as a significant source of environmental pollution with adverse effects on the reproduction and fetal development of aquatic organisms [52]. Aspirin concentrations in wastewater vary across locations, with measurements ranging from 0.4–0.7 µg/L in India [53] to 7.3 µg/L in Japan [54]. *Yersinia enterocolitica* laccase, expressed in *Escherichia coli*, demonstrated the complete biotransformation of aspirin within 24 h at pH 9.0 [40]. Al-sareji et al. [55] used laccase from *Trametes versicolor* (Sigma-Aldrich, St. Louis, MO, USA) and observed a 72% removal of aspirin after 6 h of enzymatic treatment. FTIR analysis of aspirin under various pH conditions revealed changes in the stretching of C=C bonds in the benzene ring and alternations in R-C=O bonds after 1 h of incubation (Figure 3).

Ketoprofen (2-(3-benzoylphenyl)propionic acid), a nonsteroidal anti-inflammatory drug used to treat muscle and joint pain, along with conditions such as arthritis, gout, and rheumatoid osteoarthritis, has been found at concentrations as low as 0.16 ng/L in Italy [56] or as high as 260 μg/L in India [53]. The increasing concern arises from the presence of enantiomers in the racemic mixture used in pharmaceutical formulations and potential ecotoxicological effects of transformation products on various organisms, including vertebrates, invertebrates, plants, and microorganisms [57]. *T. versicolor* laccase (Sigma-Aldrich) demonstrated the removal of 70% of ketoprofen within a 6-h reaction [55]. The primary focus of the study was conducted into the reaction mechanisms of the toxicity of degradation or biotransformation products after the enzyme-catalyzed reaction.

**Figure 4:**
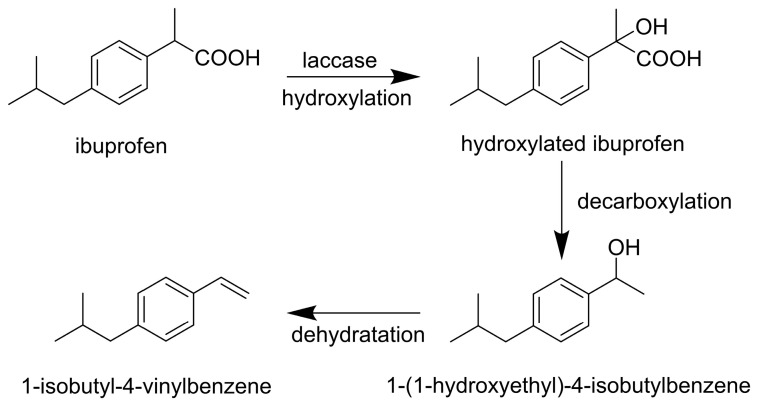
Biotransformation of ibuprofen by laccase from *T. versicolor* [62]. Copyright (2024) with permission from Elsevier.

Ibuprofen (2-methyl-4-[2-methylpropyl]phenylacetic acid), the third most widely used non-steroidal anti-inflammatory drug globally [58], is employed to alleviate pain and treat inflammatory diseases. Its concentration in the water system ranges from 3.5 to 2200 mg/L, depending on the location [59,60], posing a potential hazard to human health [61]. In the study conducted by Zhang et al. [62], *T. versicolor* laccase (Spectrum Labs) removed 76% of ibuprofen in 8 h at an initial concentration of 2.5 mM determined via HPLC. The kinetic parameters of the laccase-catalyzed ibuprofen removal reaction were Km 6.21 mM and Vmax 2.56 M/h. Biotransformation products were analyzed via GC-MS in the positive ion mode and FTIR, revealing a putative pathway involving the hydroxylation of ibuprofen, decarboxylation of hydroxylated ibuprofen, and subsequent dehydration (Figure 4).

Despite the diverse laccase producers examined in the biotransformation of analgesics (aspirin, ketoprofen, and ibuprofen), there is potential for their application in WWTPs as they demonstrated efficacy at relatively high temperatures (35–45 °C) and within a pH range of 4.0–9.0. Laccase alone proved to be sufficiently efficient in biotransforming this group of drugs (refer to Table 2), although, thus far, only laccases with a high redox potential produced by fungi have been investigated (refer to Table 1). 

### 3.2. Antibiotics

Antibiotics constitute one of the most widely prescribed and used drug categories globally. Due to their extensive usage and limited metabolism within the human body (with up to 70% excretion in an unchanged form), their release into the environment is steadily increasing, leading to elevated concentrations in water, soil, and sediments [63]. In raw wastewater (including hospital effluents) and treated wastewater, antibiotic concentrations exhibit a wide range, from a few ng/L to several tens of mg/L [64]. The rise of antibiotic resistance in clinically significant bacterial species poses a global public health challenge, necessitating the essential monitoring of antibiotic contamination in the environment. 

Laccase efficacy has been extensively examined for various antibiotic classes, including sulfonamides, penicillins, fluoroquinolones, tetracyclines and others like trimethoprim. Antibiotics have been the focus of intensive studies concerning laccase, resulting in several reviews on this topic [30,65]. These studies encompass evaluations with crude laccase extracts or the cultivation of laccase-producing organisms in media containing antibiotics. In a study conducted by Becker et al. [66], the degradation of 38 antibiotics by laccase from *T. versicolor* (Sigma-Aldrich) was investigated, and their concentrations were assessed after the enzymatic reaction using HPLC. The efficiency of biotransformation varied based on the structural characteristics of the compounds. Notably, penicillins, such as amoxicillin (96.6%) and ampicillin (88.6%), fluoroquinolones (e.g., ofloxacin, ciprofloxacin, enrofloxacin, danofloxacin, and marbofloxacin) with removal rates ranging from 50.1 to 59.4%, and tetracyclines (oxytetracycline, chlortetracycline, doxycycline, and tetracycline) with removal rates ranging from 26.0 to 48.4%, were efficiently removed. The presence of the laccase mediator system (LMS) using syringaldehyde (SYR) increased the percentage of removal of most antibiotics to over 90%. However, the application of this mediator heightened the ecotoxicity of degradation products after the enzyme-mediated reaction, as indicated by a growth inhibition test with antibiotic-sensitive *Bacillus subtilis* and the Microtox test with *Vibrio fischeri*. It is noteworthy that laccase alone in a solution with antibiotics did not induce a similar increase in ecotoxicity. SYR likely forms derivatives in the presence of laccase, negatively impacting test organisms [66].

Sulfonamide antibiotics are widely used to treat livestock diseases and account for a high proportion of total antibiotic consumption worldwide. Laccase itself was not effective in the removal of sulfonamide antibiotics [49,66,67]. Sulfadimethoxine and sulfamonomethoxine were efficiently removed by the LMS using laccase from the white-rot fungus *Perenniporia* strain TFRI 707 and 2,2′-azino-di (3-ethyl-benzothiazoline sulfonic acid) (ABTS) or violuric acid (VA) as a redox mediator. Laccase from *T. versicolor* (Sigma-Aldrich) efficiently degraded 12 sulfonamide antibiotics with SYR (1 mM) [66]. In a study conducted by Alharbi et al. [68], the ability of *T. versicolor* laccase (Sigma-Aldrich) to degrade sulfamethoxazole at a concentration of 5 mg/L was tested, observing only 48% removal within 48 h. Shi et al. [49] observed 100% removal of the sulfonamide antibiotics sulfadiazine, sulfamethazine, and sulfamethoxazole by the LMS with SYR (1 mM) but no removal with laccase from *Echinodontium taxodii* itself. Weng et al. [67] found that deaniline and oxidative coupling are two detectable pathways for the conversion of sulfonamide antibiotics by the LMS. The coupling reaction of sulfonamide antibiotics with redox mediators was also determined via LC-MS in a study conducted by Shi et al. [49]. They observed the formation of 2,6-dimethoxybenzoquinone (2,6-DMBQ) with the tested sulfonamide antibiotics, where 2,6-DMBQ was formed after the oxidation of the natural mediators acetosyringone, SYR or syringic acid (SA) by *E. taxodii* laccase. A toxicity evaluation of the degradation products after treatment with *T. versicolor* laccase using the bioluminescent *Photobacterium leiognathi* (BLT-Screen) showed no toxic effect [68]. Furthermore, the degradation products of sulfonamide antibiotics did not have a negative effect on the growth of *Staphylococcus aureus* and *E. coli* [49], confirming the biotransformation of the antibiotic to a form without the original antibiotic effect.

Penicillins represent one of the most extensively used classes of antibiotics, primarily owing to their broad spectrum of clinical applications. Depending on their structure, penicillins can serve as substrates for laccase produced by both bacteria [69] and fungi [66]. The laccase from *T. versicolor* (Sigma-Aldrich) demonstrated efficient removal of amoxicillin and ampicillin (96.6 and 88.6%, respectively). Additionally, penicillin G, penicillin V, cloxacillin, and oxacillin were degraded using the LMS with SYR as a mediator at a concentration of 1 mM, resulting in removal rates ranging from 53.5 to 93.9% [66]. Moreover, laccase from *B. subtilis*, expressed in *E. coli*, exhibited the complete removal of ampicillin within two hours, as confirmed via HPLC analysis [69]. The authors subsequently proposed a degradation mechanism featuring two putative pathways, wherein the cleavage of the β-lactate ring occurs during ampicillin degradation by laccase, leading to the subsequent loss of antibiotic activity. The degradation mechanism was corroborated by ecotoxicity assays utilizing *B. subtilis* and *E. coli*.

**Figure 5:**
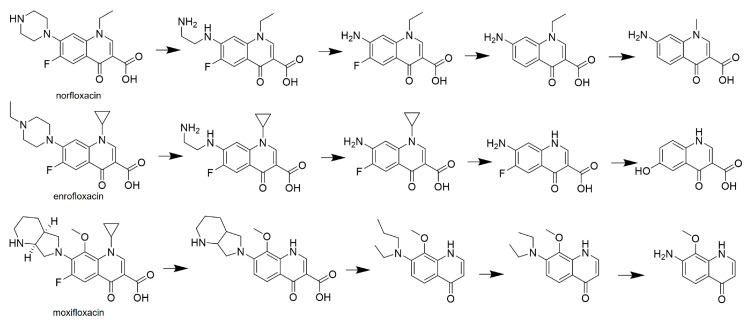
Biotransformation of fluoroquinolones by laccase from *T. versicolor* [70]. Copyright (2024) with permission from Elsevier.

Fluoroquinolones represent a substantial group of broad-spectrum bactericidal agents, characterized by a common bicyclic nuclear structure related to 4-quinolone and are employed in the treatment of bacterial infections. In a study conducted by Zou et al. [70], the efficacy of laccase from *T. versicolor* (Sigma-Aldrich) in degrading fluoroquinolones, specifically norfloxacin, enrofloxacin, and moxifloxacin, was investigated. The laccase was immobilized in a magnetically modified biochore, primarily facilitating antibiotic removal through sorption—a common phenomenon observed with immobilized laccases [71,72]. While the presence of laccase slightly increased the removal percentage (by 0.7–2.6%), the addition of ABTS as a redox mediator further enhanced removal by 11.0–16.8%. Additionally, the removal of ciprofloxacin by laccase from *T. versicolor* (Sigma-Aldrich) was found to be effective in the presence of a redox mediator, either *p*-coumaric acid (*p*-CA) (57%) or HBT (81%). It is noteworthy that a higher percentage of removal was observed in the phosphate buffer compared to the wastewater sample (approximately 1.6 times less) [71]. The proposed putative biotransformation mechanism for fluoroquinolones by laccase was elucidated through LC-MS analysis (Figure 5).

The typical biotransformation mechanism of fluoroquinolones involves processes such as the loss of ethyl/ethylene, defluorination, and demethylation or decarboxylation (Figure 5). In a study conducted by Zou et al. [71], the laccase-mediated pretreatment of ciprofloxacin resulted in only a negligible inhibition of *B. subtilis*, *S. aureus,* and *Pseudomonas aeruginosa*. This observation affirms the positive impact of laccase in the biotransformation of antibiotics.

**Figure 6:**
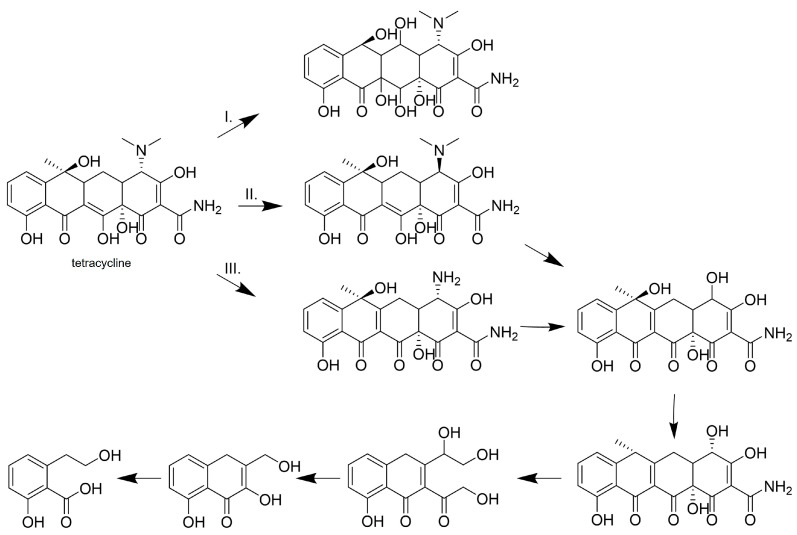
Biodegradation of tetracycline by laccase from *B. amyloliquefaciens* [72]. The possible pathways of tetracycline biotransformation by laccase are designated as I., II. and III. Copyright (2024) with permission from Elsevier.

Tetracycline antibiotics as broad-spectrum antibiotics are widely used to treat human and animal diseases, making them the second most widely used antibiotics in the world [73]. Laccase produced by bacteria and expressed in *E. coli,* as well as laccase from *Myceliophthora thermophila*, expressed in *Aspergillus* sp., have been tested [72,74]. Laccase from *Bacillus amyloliquefaciens*, expressed in *E. coli*, was able to efficiently remove tetracycline antibiotics such as tetracycline, doxycycline, and tigecycline with the efficiency of 86.1, 96.5 and 81.0%, respectively [72]. Tetracycline was biotransformed to a level below the detection limit of the HPLC analysis after a 2-h catalyzed reaction with laccase of *B. subtilis*, expressed in *E. coli* [69]. Laccase from *M. thermophila* exhibited the complete removal of tetracycline using SYR as a redox mediator even in salt water [74]. The authors proposed a putative mechanism of tetracycline biotransformation with two possible pathways, both leading to the opening of the tetracycline ring to form small acid molecules, resulting in a loss of antibiotic activity. In the case of tetracycline in the presence of laccase from *B. amyloliquefaciens*, the opening of the aromatic ring was observed in two of the three putative pathways, preceded by epimerization, demethylation, deamination, dehydrogenation, and hydroxylation in the second putative pathway or demethylation and dehydrogenation, dehydroxylation in the third pathway (Figure 6) [72]. 

The same reactions, leading to the formation of degradation products with a smaller molecular size, have also been observed for tetracycline degraded by fungal laccase [74] and other tetracycline antibiotics such as doxycycline and tigecycline [72]. The loss of the antibiotic effect of tetracycline was also confirmed by ecotoxicity assays determined via the incubation of degradation products with *B. subtilis* and *E. coli* bacteria [69]. 

Trimethoprim (diaminopyrimidine) is an antibiotic used for treating and preventing urinary tract infections. In a study conducted by Alharbi et al. [68], the efficacy of laccase from *T. versicolor* (Sigma-Aldrich) in degrading trimethoprim at a concentration of 5 mg/L was investigated. The results demonstrated a 95% removal of this drug within 48 h. LC-MS was used in the study to identify degradation products, but none were detected in either the positive or negative scanning mode. Furthermore, testing against the bioluminescent *P. leiognathi* (BLT-Screen) after the enzyme-catalyzed reaction revealed no toxic effect, indicating that the laccase-catalyzed reaction did not lead to the formation of toxic products [68]. 

In summary, it can be concluded that bacterial or fungal laccase, without a redox mediator, exhibited efficient removal of penicillins, fluoroquinolones, and tetracyclines, with degradation involving the opening of the antibiotic’s aromatic rings. The degradation percentage varied based on the antibiotic’s structure and the presence of electron-donating or electron-withdrawing functional groups (Table 3). An enhanced degradation efficiency was observed with the use of the LMS employing SYR, HBT, or ABTS [66,67,71,74]. Sulfonamide antibiotics proved to be the most resistant to laccase-catalyzed degradation, requiring the presence of a redox mediator for a decrease in concentration. Putative degradation pathways indicated biotransformation through crosslinking with a redox mediator [49,67]. Importantly, the antibiotic effect was eliminated in all studied cases, but the choice of a mediator in the LMS is crucial to avoid the production of toxic products [69,70,72,74].

### 3.3. Antiepileptics

Carbamazepine is a pharmaceutical drug that is known mainly as an antiepileptic or anticonvulsant that is prescribed to relieve neurological pain. It is one of the pharmaceutically important compounds frequently present in wastewater, with levels reaching, for example, 356.1 ± 5.8 ng/L in Canada [5] or 870 ng/L in the Netherlands [4]. It is toxic to the aquatic environment [75]. Laccase from *M. thermophila,* expressed in *Aspergillus* sp. (Sigma-Aldrich), was used to remove carbamazepine. The authors found that the highest percentage of drug removal (46%) assessed via HPLC analysis was observed at pH 7.0 and a temperature of 25 °C, with Vmax of 0.873 ± 0.005 mg/L/h and Km of 28,827 ± 0.060 mg/L within 24 h [76]. Laccase isolated from *T. versicolor* (Sigma-Aldrich) was able to remove 82% carbamazepine within 48 h at an initial concentration of 5 mg/L (Table 4) [68]. In a study conducted by Ji et al. [14], laccase from *T. versicolor* (Sigma-Aldrich) removed only 5% of carbamazepine. The difference between these two studies lies in the use of a different pH (Table 4).

**Figure 7:**
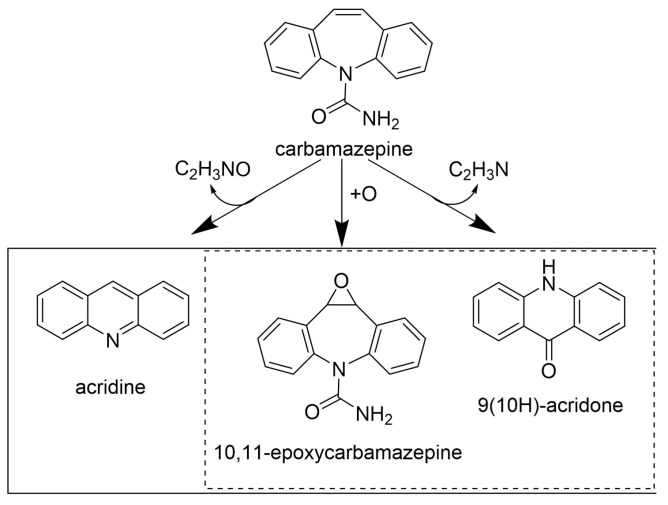
Carbamazepine biotransformation metabolites identified via LC-MS (modified by [76]). Copyright (2024) with permission from Elsevier.

The use of an LMS with *p*-CA as a redox mediator resulted in a carbamazepine removal of 60%. Partially purified laccase produced by *T. versicolor* IFO-6482 with HBT as a redox mediator was used for carbamazepine degradation [77]. The enzyme preparation was added to the mixture six times during the enzyme reaction at 8-h intervals. The percentage of drug removal was 39% for laccase itself and 60% for the LMS after 48 h. The formation of acridine, 9(10H)-acridone, and 10,11-epoxycarbamazepine was observed after the enzyme-catalyzed reaction via LC-MS [76]. The presence of 11-epoxycarbamazepine and 9[10H]-acridone has been confirmed in other studies [68,77] (Figure 7).

The presence of additional compounds was also determined when the LMS with the natural mediator *p*-CA was used. These products were formed by the coupling of carbamazepine with various oxidative forms of *p*-CA molecules and the radical condensation products of this mediator due to the formation of 5-5′ and β-β′ bonds. However, the formation of these dimers, oligomers and even polymers can be an advantage in the removal of pharmaceuticals in WWTPs. They can be removed via precipitation followed by sedimentation or filtration or by physical adsorption onto other components such as biomass also present in wastewater [14]. The toxicity of these biotransformation products was evaluated against luminescent *P. leiognathi* [68] and *V. fisheri* [78], as well as algal viability [14], and the results revealed that the investigated metabolites had no toxic effect. Carbamazepine contains an amide as an electron-withdrawing functional group, which reduces the susceptibility of this substrate to laccase degradation [68,76]. However, the high redox potential of fungal laccase (Table 1) itself was able to biotransform 82% of this compound under appropriately adjusted reaction conditions [68]. The enzymatic treatment of carbamazepine appears to be a more efficient and environmentally acceptable method than the removal of this drug via ozonation or UV photolysis. In these processes, the formation of a higher number of degradation products (7 and 13 for ozonation and UV photolysis, respectively) with toxic effects was observed [9,10] compared to laccase pretreatment [68].

### 3.4. Antirheumatics

Naproxen and diclofenac, belonging to the class of non-steroidal anti-inflammatory drugs that inhibit prostaglandin synthesis, have been found in rivers and wastewater, raising concerns for aquatic systems [75]. Worldwide, naproxen concentrations in rivers ranged from 0.04 to 32 μg/L [79], while diclofenac in wastewater in the Netherlands reached concentrations of up to 280 ng/L [4].

**Figure 8:**
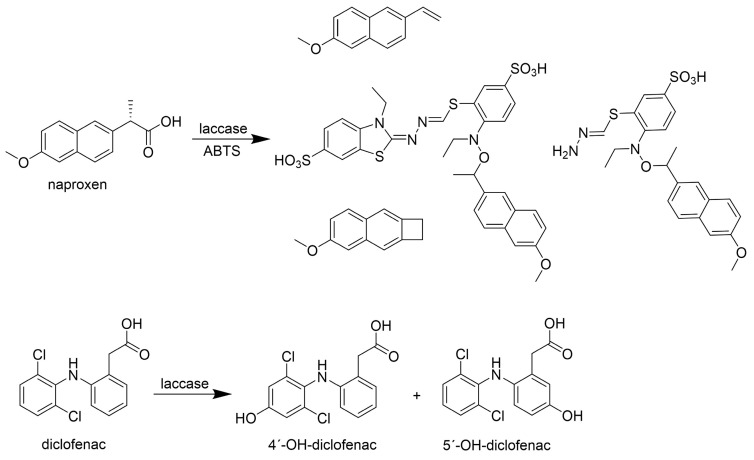
Naproxen and diclofenac biotransformation metabolites identified via LC-MS adapted by [40,80]. Copyright (2024) with permission from Elsevier.

Naproxen exhibits relative resistance to laccase-catalyzed degradation, often necessitating the use of an LMS [44,80]. A study using the LMS with laccase from *M. thermophila,* expressed in *Aspergillus* sp. (Novozymes), and HBT or violuric acid (VA) as redox mediators demonstrated a removal percentage of 68% after 24 h with HBT [44]. The enzyme reaction was carried out at pH 4.0, deemed optimal for the enzyme but potentially challenging for WWTPs with a pH ranging from 6.0 to 8.0. Another study with laccase from *T. versicolor* (Sigma-Aldrich) using ABTS as a redox mediator achieved a remarkable 95% removal of naproxen after 24 h [80]. The degradation process of naproxen via the LMS with ABTS as a redox mediator resulted in the elimination of formic acid from naproxen, yielding three different products, with the main product identified as 1-(6-methoxynaphthalen-2-yl)ethanone (Figure 8).

The formation of carbamazepine-like conjugation products was also observed [14]. The disadvantage of the formation of these conjugation products is a decrease in the concentration of the mediator due to its incorporation into the conjugation product. The products of the laccase–ABTS treatment showed a lower anti-inflammatory effect against *Artemia salina* growth than naproxen [80].

Diclofenac is effectively eliminated by laccase [40,44,48,80,81,82]. Diclofenac was completely removed by laccase from *T. versicolor* (Sigma-Aldrich) within 12 h [68] or 95% within 24 h [80]. Laccase from *M. thermophila,* expressed in *Aspergillus* sp. (Novozymes), exhibited the complete removal of diclofenac within 8 h and the LMS using HBT, SYR, or VA as redox mediators within 1 h [44]. Laccase from *Y. enterocolitica,* expressed in *E. coli,* showed 100% biotransformation of diclofenac after 24 h [40]. The least efficient degradation of diclofenac was by laccase from *Moniliophthora roreri,* expressed in *Pichia pastoris*, resulting in 58% removal after 24 h [48] (Table 5).

The degradation products were identified via LC-MS analysis [68,81]. One of them was formed by hydroxylation of the benzene ring (4-hydroxy derivative of diclofenac) with two chlorine atoms and was found to be formed in greater amounts compared to the second identified product (5-hydroxy derivative of diclofenac), which was formed by hydroxylation at the *p*-position of the amino group of the benzene ring [86]. However, after the degradation of diclofenac, both products were no longer detected in the mixture, indicating that they are highly degradable by laccase [68]. Only diclofenac daughter ions due to incomplete degradation were identified after 24 h [80]. The chemical structure of diclofenac contains an aromatic amine that belongs to an electron-donating functional group, which contributes to the degradation of the drug by laccase [87]. The same degradation products were also observed after the reaction with laccase from *Y. enterocolitica* [40], but their presence was detected after 24 h. The FTIR results further confirm the diclofenac transformation and suggest a change in C-C and C=C bonds [40].

Laccase produced by *Sclerotinia sclerotiorum* [82] formed the same products as reported by other authors [68,80], but the presence of a precipitate was observed. The precipitate contained high-molecular-weight oligomers, making this enzymatic method of diclofenac treatment applicable in WWTPs. The BLT-Screen of the sample after 48 h of diclofenac with laccase from *T. versicolor* (Sigma-Aldrich) showed a non-toxic effect, which means that laccase does not cause the formation of toxic products [68], unlike the removal of diclofenac via ozonation [9] or UV photolysis [10]. Like other drugs, enzymatic biotransformation appears to be a more environmentally friendly alternative than other drug removal methods. In addition, the formation of conjugates with a high-molecular-weight size was observed for both drugs tested, which will facilitate their subsequent isolation from wastewater.

### 3.5. Cytostatics

Cytostatics are drugs designed to inhibit the growth of tumor cells or destroy tumor-altered tissues in the body. Despite their anticancer properties, these drugs are not tumor-specific and may exhibit cytotoxic, mutagenic, teratogenic, or carcinogenic effects even at low concentrations [8,88]. They have been identified in wastewater near hospitals at concentrations ranging from 0.02 to 0.025 μg/L [7], and their removal in WWTP is not consistently effective [8,88,89,90]. The effectiveness of laccase was evaluated using doxorubicin and etoposide as model anticancer drugs [83,84,85].

Doxorubicin is a widely used anticancer drug for the treatment of various types of cancer. In both studies that dealt with the elimination of this drug from a solution, laccase from *T. versicolor* (Sigma-Aldrich) was used [83,84]. In both cases, either fluorescence spectrophotometry (λ_ex_ = 480 nm, λ_em_ = 598 nm) [83] or VIS spectrophotometry (λ = 480 nm) [84] was used to determine the residual drug concentration. Kelbert et al. [83] found that the highest percentage of removal was observed at pH 7.0 and a temperature of 30 °C, with a Vmax of 702.8 μg/h/L and Km of 4.05 μM, indicating a strong affinity of the enzyme for the substrate. In a study conducted by Jinga et al. [84], the efficiency of doxorubicin elimination from a solution using the LMS with TEMPO (2,2,6,6-tetramethylpiperidin-1-yl)oxyl) as a mediator was evaluated. The complete elimination of doxorubicin was observed at a concentration of 50 mg/L at pH 7.0 for 24 h. Kelbert et al. [83] tested the cytotoxic effect by altering the murine fibroblast cell line (L-929). Doxorubicin showed a cell viability of 51.2 ± 2.6% at the highest concentration used (1 mg/L), while the degradation products after the enzyme-catalyzed reaction at an initial concentration of 1 mg/L showed 72.9 ± 3.2%. This implies that there was a decrease in the cytotoxic effect after the enzyme-catalyzed reaction. A drawback of both of the above papers is the absence of LC-MS analysis and the indication of the laccase action mechanism on the drug, so it is not possible to say whether there was degradation of the drug or only biotransformation to a less toxic form.

Etoposide is a drug that is used in the treatment of various cancers. Like doxorubicin, it was detected in wastewater around hospitals at concentrations of 6 to 380 ng/L (China) or 714 ng/L (Spain) [89,91]. Its presence in wastewater poses a threat due to its potential carcinogenicity, mutagenicity, and genotoxicity [92]. Pereira et al. [85] tested the ability of laccase from *T. versicolor* (Sigma-Aldrich) to remove this drug. Laccase with a high enzyme activity of 1100 U/L achieved 100% degradation within 1 h, while reducing the laccase activity to 55 U/L achieved 86% removal of etoposide within 6 h, with approximately 68% removal after 1 h. Dark-brown products after the enzyme-catalyzed reaction were determined via LC-MS-MS, with results indicating a biotransformation of the colorless drug via dehydration and demethylation. Compared to the original drug, the products of laccase biotransformation were non-cytotoxic to the murine fibroblast cell line (L-929).

In both cases, laccases were effective under conditions that are achievable in WWTPs, such as a pH in the range of 6.0–7.0 and a temperature of approximately 30 °C, indicating their potential commercial use in the removal of these compounds from wastewater.

### 3.6. Hormones

The hormones estrone (E1), 17β-estradiol (E2), and estriol (E3) as natural estrogens, along with ethinylestradiol (EE2) as a synthetic estrogen, belong to the group of estrogenic compounds used in the hormonal treatment of menopausal symptoms. The content of estrone [E1] and 17β-estradiol [E2] in deep groundwater was measured at 68.1 and 2.5 ng/L, respectively [93]. The presence of estrogens poses a problem for the aquatic ecosystem and has been associated with abnormal sexual differentiation or reproductive disorders in wild fish [94].

Laccase alone efficiently removes hormones such as E2, E3, and EE2 [44,48,95,96]. For estrone E1, the use of the LMS is required [44] (Table 6). Laccase from *M. thermophila,* expressed in *Aspergillus* sp. (Novozymes), removed 100% of E2 within 3 h and E22 within 5 h. E1 was removed with 65% of efficiency within 24 h, and in the presence of the LMS using VA, the removal was 100% [44]. A short time (0.5 h) was also required to remove E2, E3 and EE2 by laccase from *M. roreri* expressed in *P. pastoris* [48]. As in the previous study, E1 was the most resistant to laccase degradation, with a residual concentration of 55% after 1 h, but extending the time to 20 h resulted in a concentration below the detection limit of the HPLC method. E2 was also removed by laccase from *T. versicolor* (Sigma-Aldrich), achieving 95.3% removal in 5 h [96]. The same enzyme from *T. versicolor* (Sigma-Aldrich) was used to remove E2 and EE2 with 92 and 100% efficiency after 24 h of incubation, respectively [97]. Laccase from *M. thermophila* expressed in *Aspergillus oryzae* (Novozymes) removed 93–98.5% of E1, E2, E3 and E22 within 72 h [98].

Zdarta et al. [97] analyzed a mixture containing E2 or EE2 with laccase and did not observe the formation of a precipitate containing dimers or oligomers, as observed by other authors [99,100], although in all studies, laccase from *T. versicolor* (Sigma-Aldrich) was used, and the conditions of the enzyme-catalyzed reaction were comparable (25 °C, pH 5.0). The absence of a precipitate, along with the significant reduction in total organic carbon and total carbon, as well as the formation of CO_2_ and water during biodegradation suggests the mineralization of both drugs as the main mechanism of the enzyme reaction [97]. Mineralization occurred due to the oxidation of both estrogens followed by a cleavage of C-C bonds, ring openings, and further rearrangement in the estrogen structure to form low-molecular-weight inorganic compounds as the main products of the laccase-assisted biodegradation of E2 and EE2. The estrogen degradation products after the laccase-catalyzed reaction exhibited low estrogenic activity (98.75% for E2 and 86.81% for E22). Therefore, enzymatic degradation is a highly efficient method for removing estrogens from aqueous solutions, either via precipitate formation or the complete mineralization of the drugs, and the resulting bioconversion products are significantly less toxic to living organisms than the original estrogen solutions.

### 3.7. Anxiolytics, Sympatholytics

Anxiolytics, such as benzodiazepines, are widely prescribed medications for anxiety and insomnia treatment [101]. However, their extensive use contributes to environmental contamination with wastewater levels ranging from 3 to 87 ng/L in Germany and Spain [102,103].

Seven benzodiazepines, including alprazolam, chlordiazepoxide, lorazepam, diazepam, clobazam, oxazepam, and nitrazepam, were subjected to degradation at a concentration of 10 mg/L using purified laccase from *Paraconiothyrium variabile*. After 48 h, nitrazepam, alprazolam, diazepam, and oxazepam concentrations decreased by 27.3, 45.6, 18.6 and 18.7%, respectively. However, clobazam, chlordiazepoxide, and lorazepam exhibited resistance to degradation, even when the LMS with 2 mM HBT as a redox mediator was applied. Improved drug removal was observed using the LMS for other benzodiazepines: nitrazepam (73%), alprazolam (88.1%), diazepam (61.4%) and oxazepam (71.2%) [104]. Additionally, diazepam underwent degradation with laccase from *T. versicolor* (Fluka), resulting in a 68% removal after 12 h. The presence of the redox mediator HBT did not significantly enhance removal [105]. However, the degradation mechanism and the toxic effect of the mixture post enzyme-catalyzed reaction have not been described yet.

Sympatholytics, including β-blockers, constitute a crucial drug subclass widely used for treating cardiac arrhythmias and hypertension. The presence of β-blockers has been detected in effluents at concentrations with units ranging from of ng/L to μg/L [106,107]. Similar to other pharmaceuticals, their presence in the aquatic ecosystem poses significant harm to organisms such as fish, phytoplankton and daphnia [108,109,110].

Among the β-blockers, atenolol has been tested as a potential substrate for laccase [105,111]. In a study using laccase from *T. versicolor* (Fluka), up to 90% removal of atenolol was achieved within 12 h. Interestingly, the addition of a redox mediator (HBT) at a concentration of 2 mM did not significantly enhance the removal percentage [104]. Feng et al. [111], using a commercially purchased laccase from *T. versicolor* (Sigma-Aldrich), observed that the enzyme alone did not reduce the concentration of atenolol within 24 h. Effective removal occurred only in the presence of redox mediators, particularly TEMPO. However, the use of TEMPO as a mediator has a disadvantage due to its toxicity [111]. The combination of atenolol and TEMPO resulted in 95% lethality of zebrafish embryos within 72 h, highlighting the toxic effects associated with this mediator.

## 4. Conclusions and Future Perspectives

The potential for biotransformation or even biodegradation to mineralization depends on two factors: the structure of the enzyme, determined by the producer, and the structure of the degraded compound. Since the structure of the degraded compound remains unchanged, enhancing the efficiency of biodegradation for pharmaceutical products involves modifying the enzyme’s structure. Advances in modern biotechnology, protein engineering, and bioinformatics enable the modification of enzymes, leading to the design of more efficient enzyme variants. However, achieving this goal requires understanding the molecular-level structural modifications essential for enhancing drug degradation. The use of only a few structurally distinct enzymes in pure form for drug degradation (Table 1) prevents defining precise requirements for enzyme structure changes. To optimize the degradation of pharmaceuticals and other anthropogenic organic pollutants, it is crucial to explore enzymes produced by different microorganisms and define the structural interventions needed for efficient direct degradation by laccase.

In addition to the direct degradation of organic matter by laccase, the LMS can be employed. However, a drawback is the potential formation of substances exhibiting toxic effects on organisms [14,82,97]. While certain studies have demonstrated toxic effects [60,66,69], the key lies in evaluating the final form of degradation products. The LMS operates through the transfer of oxidative potential, forming reactive species, mainly radicals, which can revert to their original form. Radicals and other compounds can also conjugate to create dimers, oligomers, and polymers of these substances [34,35]. The immediate evaluation of an enzymatic reaction after a laccase-catalyzed reaction may still show the presence of radicals, acting on organisms by a similar mechanism and inhibiting their growth. Achieving a more efficient system requires a deeper understanding of the chemical mechanisms of these reactions, considering the chemical structure of the mediator and the substance being degraded.

Given that pharmaceutical degradation primarily occurs in wastewater with low concentrations of substances, utilizing immobilized enzymes has become necessary. Flow-through columns with immobilized enzymes and membrane systems that selectively permit certain substances to pass through have been proposed [14,49,55,76]. However, defining the technological parameters of these processes necessitates a deeper understanding of laccase immobilization techniques, along with their integration into membrane systems. Challenges in this realm include the potential loss of catalytic activity, enzyme release from the immobilization matrix, limitations in mass transfer, and the cost associated with immobilization matrices [71,72]. The development of these technologies requires an evaluation of their economic viability, ensuring the long-term sustainability of the removal of pollutant removal from the environment through laccase.

## Figures and Tables

**Table 1:** Characterization of laccases used for the removal of pharmaceutical products.

Producer	Redox Potential (V vs. NHE)	Biochemical Properties			Ref.
		pH	Temperature (°C)	Kinetic Parameters	
*Bacillus subtilis* [B]	0.440	7.0	55	Km = 2070 μM	[36,37]
				Vmax = 6500 U/mL	
				(SYR)	
*Bacillus amyloliquefaciens* [B]	n.d.	4.0	65	Km = 436.8 μM	[38,39]
				(ABTS)	
*Yersinia enterocolitica* ^a^ [B]	0.27–0.432	9.0	70	Km = 2070 μM	[40,41]
				Vmax = 6500 U/mL	
				(guaiacol)	
*Sclerotinia sclerotiorum* [F]	n.d.	4.0	60–70	Km = 85.8 μM	[42]
				Vmax = 18.64 U/mL	
				(ABTS)	
*Myceliophthora* *Thermophila* ^b^ [F]	0.460	4.0	50	Km = 52.151 μM	[43,44,45,46]
				Vmax = 11.493 mU	
				(ABTS)	
*Paraconiothyrium variabile* [F]	n.d.	4.8	50	Km = 203 μM	[47]
				Vmax = 40 U/mg	
				[ABTS]	
*Moniliophthora roreri* ^c^ [F]	0.58	4.0	n.d.	Km = 24.13 μM	[48]
				(ABTS)	
*Echinodontium taxodii* [F]	n.d.	3.0	60	Km = 41.4 μM	[49]
				Vmax = 5.9 U/mL	
				(ABTS)	
*Trametes versicolor* [F]	0.990	4.0	40	Km = 297 μM	[29,50]
				Vmax = 26.96 U/mg	
				(ABTS)	

Recombinant laccase expressed in ^a^ *Escherichia coli*, ^b^ *Aspergillus* sp. or ^c^ *Pichia pastoris*. ABTS—2,2′-azino-di(3-ethyl-benzothiazoline sulfonic acid), B—bacteria, n.d.—not determined, F—fungi, NHE—normal hydrogen electrode, SYR—syringaldehyde.

**Table 2:** Laccases and their use in biotransformation of analgesics.

Drug	Concentration [mg/L]	Laccase Producer	Laccase Activity [U/L]	Enzyme Reaction	Degradation/Transformation Efficiency *	Ref.
Aspirin	5.0	*Yersinia enterocolitica*	100	45 °C, pH 9.0, 24 h	100% (BT)	[40]
	25.0	*Trametes versicolor*	40	35 °C, pH 4.0, 6 h	72% (n.s.)	[55]
Ketoprofen	25.0		40	35 °C, pH 4.0, 6 h	70% (n.s.)	
Ibuprofen	515.7		29	40 °C, pH 7.0, 8 h	76% (BT)	[62]

* BT—biotransformation, n.s.—not specified.

**Table 3:** Laccases and their use in degradation/ biotransformation of antibiotics.

Drug	Concentration [mg/L]	Laccase Producer	Laccase Activity [U/L]	Concentration of Redox Mediator [mM]	Enzyme Reaction	Degradation/Transformation Efficiency	Ref.
Sulfamonomethoxine	50	*Perenniporia* TFRI 707	600	ABTS [1.0]	30 °C, pH 4.0, 0.5 h	100% (BT)	[67]
				VA [1.0]		100% (BT)	
	0.01	*Trametes versicolor*	n.s.	-	25 °C, pH 7.0, 24 h	5.4% (n.s.)	[66]
				SYR [1.0]		96.1% (n.s.)	
Sulfadimethoxine	50	*Perenniporia* TFRI 707	600	ABTS [1.0]	30 °C, pH 4.0, 0.5 h	100% (BT)	[67]
				VA [1.0]		100% (BT)	
Sulfadiazine	0.01	*Trametes versicolor*	n.s.	-	25 °C, pH 7.0, 24 h	11.2% (n.s.)	[66]
				SYR [1.0]		99.7% (n.s.)	
	50	*Echinodontium taxodii*	200	SA [1.0]	30 °C, pH 5.0, 0.5 h	100% (BT)	[49]
Sulfamethoxazole	50	*Echinodontium taxodii*	200	SA [1.0]	30 °C, pH 5.0, 0.5 h	100% (BT)	[49]
	5.0	*Trametes versicolor*	430–460	-	25 °C, pH 6.8–6.9, 8 h	56% (n.s.)	[68]
	0.01	*Trametes versicolor*	n.s.	-	25 °C, pH 7.0, 24 h	14.2% (n.s.)	[66]
				SYR [1.0]		97.2% (n.s.)	
Tetracycline	0.01	*Trametes versicolor*	n.s.	-	25 °C, pH 7.0, 24 h	26.0% (n.s.)	[66]
				SYR [0.01]		85.2% (n.s.)	
	100	*Bacillus subtilis*	34.3	-	25 °C, pH 5.5, 2 h	100% (D)	[69]
	100	*Bacillus amyloliquefaciens*	2000	-	30 °C, pH 7.0, 2 h	86.1% (D)	[72]
	0.002	*Myceliophthora thermophila*	200	SA [0.001]	15 °C, pH 8.0, 2 h	100% (D)	[74]
Doxycycline	100	*Bacillus amyloliquefaciens*	500	-	30 °C, pH 7.0, 2 h	96.5% (D)	[72]
	0.01	*Trametes versicolor*	n.s.	-	25 °C, pH 7.0, 24 h	30.4% (n.s.)	[66]
				SYR [0.01]		89.1% (n.s.)	
Tigecycline	100	*Bacillus amyloliquefaciens*	500	-	30 °C, pH 7.0, 2 h	81.0% (D)	[72]
Norfloxacin	0.01	*Trametes versicolor*	n.s.	-	25 °C, pH 7.0, 24 h	58.1% (n.s.)	[66]
				SYR [1.0]		82.4% (n.s.)	
Enrofloxacin	0.01	*Trametes versicolor*	n.s.	-	25 °C, pH 7.0, 24 h	50.1% (n.s.)	[66]
				SYR [1.0]		76.6% (n.s.)	
Ciprofloxacin	10	*Trametes versicolor*	n.s.	*p*-CA [3.0]	45 °C, pH 6.8, 6 h	57% (n.s.)	[71]
				HBT [3.0]		81% (n.s.)	
Amoxicillin	0.01	*Trametes versicolor*	n.s.	-	25 °C, pH 7.0, 24 h	96.6% (n.s.)	[66]
				SYR [1.0]		94.7% (n.s.)	
Ampicillin	0.01	*Trametes versicolor*	n.s.	-	25 °C, pH 7.0, 24 h	88.6% (n.s.)	[66]
				SYR [1.0]		99.9% (n.s.)	
	100	*Bacillus subtilis*	34.3	-	25 °C, pH 5.5, 2 h	100% (D)	[69]
Trimethoprim	5.0	*Trametes versicolor*	430–460	-	25 °C, pH 6.8–6.9, 24 h	95% (n.s.)	[68]
	0.01		n.s.	-	25 °C, pH 7.0, 24 h	26.6% (n.s.)	[66]
				SYR [1.0]		66.8% (n.s.)	

ATBS—2,2′-azino-di[3-ethyl-benzothiazoline sulfonic acid], BT—biotransformation, D—degradation, HBT—1-hydroxybenzotriazole, VA—violuric acid, *p*-CA—*p*-coumaric acid, SYR—syringaldehyde, SA—syringic acid, n.s.—not specified.

**Table 4:** Laccases and their use in biotransformation of carbamazepine.

Drug	Concentration [mg/L]	Laccase Producer	Laccase Activity [U/L]	Concentration of Redox Mediator [mM]	Enzyme Reaction	Degradation/Transformation Efficiency	Ref.
Carbamazepine	5.0	*Myceliophthora thermophila*	n.s.	-	LT, pH 7.0, 24 h	46% (BT)	[76]
	5.0	*Trametes versicolor*	430–460	-	25 °C, pH 6.8–6.9, 48 h	82% (BT)	[68]
	5.0		500	-	LT, pH 7.0, 96 h	5% (BT)	[14]
				*p*-CA [2.0]		60% (BT)	
	4.7		600	-	30 °C, pH 4.5, 48 h	39% (BT)	[77]
				HBT [0.2]		60% (BT)	

BT—biotransformation, LT—laboratory temperature, HBT—1-hydroxybenzotriazole, *p*-CA—*p*-coumaric acid.

**Table 5:** Laccases and their use in biotransformation of other indication groups of pharmaceuticals.

Drug	Concentration [mg/L]	Laccase Producer	Laccase Activity [U/L]	Concentration of Redox Mediator [mM]	Enzyme Reaction	Degradation/Transformation Efficiency	Ref.
Naproxen	5.0	*Myceliophthora thermophila*	2000	-	LT, pH 4.0, 24 h	-	[44]
				HBT [1.0]		68% (n.s.)	
				VA [1.0]		36% (n.s.)	
	1.0	*Trametes versicolor*	250	ABTS [0.1]	25 °C, pH 5.0, 24 h	95% (D)	[80]
Diclofenac	29.6	*Sclerotinia sclerotiorum*	456	-	LT, pH 5.0, 30 h	96% (BT)	[82]
	5.0	*Yersinia enterocolitica*	100	-	45 °C, pH 9.0, 24 h	100% (BT)	[40]
	5.0	*Myceliophthora thermophila*	2000	-	LT, pH 4.0, 8 h	100% (n.s.)	[44]
				HBT [1.0]	LT, pH 7.0, 1 h	100% (n.s.)	
	30.0	*Moniliophthora roreri*	20,000	-	LT, pH 7.0, 20 h	58% (n.s.)	[48]
	4.6	*Trametes versicolor*	10	-	LT, pH 3.0, 4 h	90% (D)	[81]
	5.0		430–460	-	25 °C, pH 6.8–6.9, 8 h	100% (D)	[68]
	1.0		250	-	25 °C, pH 5.0, 24 h	95% (D)	[80]
Naproxen	5.0	*Myceliophthora thermophila*	2000	-	LT, pH 4.0, 24 h	-	[44]
				HBT [1.0]		68% (n.s.)	
				VA [1.0]		36% (n.s.)	
Doxorubicin	0.25	*Trametes versicolor*	900	-	30 °C, pH 7.0, 2 h	100% (BT)	[83]
	25		210	TEMPO [250]	n.s., pH 7.0, 24 h	100% (n.s.)	[84]
	50					100% (n.s.)	
	75					65% (n.s.)	
Etoposide	0.5	*Trametes versicolor*	1100	-	30 °C, pH 6.0, 1 h	100% (BT)	[85]
			55			68% (BT)	

BT—biotransformation, D—degradation, LT—laboratory temperature, ATBS—2,2′-azino-di [3-ethyl-benzothiazoline sulfonic acid, HBT—1-hydroxybenzotirazole, VA—violuric acid, TEMPO—(2,2,6,6-tetramethylpiperidin-1-yl)oxyl, n.s.—not specified.

**Table 6:** Laccases and their use in degradation/biotransformation of hormones.

Drug	Concentration [mg/L]	Laccase Producer	Laccase Activity [U/L]	Concentration of Redox Mediator [mM]	Enzyme Reaction	Degradation/Transformation Efficiency	Ref.
Estrone	5.0	*Myceliophthora thermophila*	2000	-	LT, pH 4.0, 24 h	65% (n.s.)	[44]
				VA [1.0]	LT, pH 4.0, 8 h	100% (n.s.)	
	0.1		180	-	25 °C, pH 6.8 ± 0.2, 24 h	94.8% (n.s.)	[98]
	27.0	*Moniliophthora roreri*	20,000	-	LT, pH 7.0, 20 h	100% (n.s.)	[48]
17β-estradiol	5.0	*Myceliophthora thermophila*	2000	-	LT, pH 4.0, 3 h	100% (n.s.)	[44]
	0.1		180	-	25 °C, pH 6.8 ± 0.2, 24 h	98.5% (n.s.)	[98]
	27.4	*Moniliophthora roreri*	20,000	-	LT, pH 7.0, 0.5 h	100% (n.s.)	[48]
	1.0	*Trametes versicolor*	1500	-	25 °C, pH 5.0, 24 h	92% (D)	[97]
	27.4	*Trametes versicolor*	1000	-	25 °C, pH 5.0, 5 h	95.3% (n.s.)	[96]
Estriol	0.1	*Myceliophthora thermophila*	180	-	25 °C, pH 6.8 ± 0.2, 24 h	98.5% (n.s.)	[98]
	28.8	*Moniliophthora roreri*	20,000	-	LT, pH 7.0, 0.5 h	100% (n.s.)	[48]
Ethinylestradiol	0.1	*Myceliophthora thermophila*	180	-	25 °C, pH 6.8 ± 0.2, 24 h	98.2% (n.s.)	[98]
	5.0		2000	-	LT, pH 4.0, 5 h	100% (n.s.)	[44]
	5.0	*Trametes versicolor*	1500	-	25 °C, pH 5.0, 24 h	100% (D)	[97]
	29.4	*Moniliophthora roreri*	20,000	-	LT, pH 7.0, 0.5 h	100% (n.s.)	[48]

BT—biotransformation, D—degradation, LT—laboratory temperature, VA—violuric acid, n.s.—not specified.

## Abbreviations

2,6-DMBQ—2,6-dimethoxybenzoquinone; ATBS—2,2′-azino-di(3-ethyl-benzothiazoline sulfonic acid); BT—biotransformation; D—degradation; HBT—1-hydroxybenzotirazole; LMS—laccase mediator system; LT—laboratory temperature; n.d.—not determined; NHE—normal hydrogen electrode; n.s.—not specified; p-CA—*p*-coumaric acid; SA—syringic acid; SYR—syringaldehyde; TEMPO—(2,2,6,6-tetramethylpiperidin-1-yl)oxyl; VA—violuric acid; WWTP—wastewater treatment plant.
